# Systematic review with network meta-analysis: Diagnostic values of ultrasonography, computed tomography, and magnetic resonance imaging in patients with ischemic stroke

**DOI:** 10.1097/MD.0000000000016360

**Published:** 2019-07-26

**Authors:** Xiao-Hong Zhang, Hui-Min Liang

**Affiliations:** a Department of Ultrasound; b Department of Neurology, Huaihe Hospital of Henan University, Kaifeng, P. R. China.

**Keywords:** computed tomography, diffusion-weighted imaging, ischemic stroke, magnetic resonance imaging, negative predicted value, positive predicted value, sensitivity, ultrasonography

## Abstract

**Background and objective:**

Ischemic stroke is a foremost cause for disability and death worldwide. This study is conducted in order to compare the diagnostic values between transcranial Doppler ultrasound (ultrasonography), computed tomography (CT), and magnetic resonance imaging (MRI) in patients suffering from ischemic stroke by performing a network meta-analysis.

**Methods:**

We made use of Cochrane Library, PubMed, and Embase in order to obtain literature and papers. The combination analysis of both direct and indirect evidence in terms of sensitivity, specificity, positive predictive value (PPV), negative predictive value (NPV) and accuracy was conducted so as to assess the odds ratios (ORs) and surface under the cumulative ranking curve (SUCRA) values of the seven different imaging methods. These imaging techniques include ultrasonography, computed tomography (traditional CT, computed tomography angiography [CTA], computed tomography perfusion [CTP]), and MRI (traditional MRI, diffusion-weighted imaging [DWI], magnetic resonance angiography), in order to properly diagnose ischemic stroke patients.

**Results:**

Thirteen eligible diagnostic trials were enrolled into this network meta-analysis. The results of the traditional meta-analysis showed that among CT methods, CTP showed higher sensitivity, NPV, and accuracy; among MRI methods, DWI had relatively higher sensitivity, NPV, and accuracy. The results of network meta-analysis showed that DWI had relatively higher sensitivity, NPV, and accuracy when compared with traditional CT, CTA, magnetic resonance angiography and traditional MRI. CTP showed higher SUCRA among CT methods while DWI showed higher SUCRA among MRI methods. A cluster analysis revealed that DWI had the highest diagnostic value in terms of sensitivity, PPV, NPV, and accuracy amongst the aforementioned seven imaging techniques.

**Conclusion:**

This network meta-analysis provides supporting evidence to the idea that DWI has a higher diagnostic value regarding ischemic stroke among MRI methods, and CTP has a poor diagnostic value among CT methods, which provide therapeutic considerations for Ischemic stroke intervention.

## Abstract 2

Supplemental Digital Content is available in the text

## 1 Introduction

An ischemic stroke is often caused by an occlusion of the vessels located in the brain such as the middle cerebral artery, basilar artery, and carotid terminus.^[1,2]^ These incidences carry an especially high rate of mortality, as well as the survival ratio estimated between 53% and 92%.^[3,4]^ Despite the decline in the incidence of ischemic strokes over the past few decades, it remains the leading cause of death and disability in the western countries.^[5]^ Diffusion and perfusion magnetic resonance imaging (MRI) remain novel clinical imaging methods; however, the application of either one of them in the diagnosis of ischemic stroke in the acute period (first or second day) only benefits a small portion of patients.^[6]^ Therefore, in order to lessen the incidence of IS worldwide, understanding both stroke prevention and effectively treating those diagnosed are of optimal importance.^[7]^

Stroke can be diagnosed through several methods, which include; neurological examination, computed tomography (CT) scans, MRI scans, Doppler ultrasound, and arteriography. A transcranial Doppler Ultrasonography (ultrasonography) is a simple, non-invasive method that is used in order to examine the intracerebral blood flow.^[8]^ It is a safe and portable method which allows it to be utilized by the bedside in a short amount of time.^[9]^ Although various kinds of imaging methods have made great advancements since their inception, CT remains the most common and effectively used imaging method for both the ability to diagnosis acute ischemic stroke and differentiates from hemorrhagic stroke due to its lower cost, widespread availability, rapid speed, and accuracy in comparison with other major imaging methods.^[10,11]^ Computed tomography angiography (CTA) is used to examine blood vessels in the brain, kidneys, pelvis and the lungs.^[12]^ Computed tomography perfusion (CTP) is another useful imaging method which can provide significant information about the capillary-level hemodynamics of the brain parenchyma.^[13]^ MRI scans allow doctors to obtain images of diffusion and perfusion, and has been shown to be very useful in the diagnosis of acute ischemic stroke, while comparatively differentiating it from hemorrhagic strokes.^[14]^ Magnetic resonance angiography (MRA) is commonly used in order to evaluate the status of both cervical and intracranial arteries as well as determine the presence of ischemic tissue at risk, which has been used in comprehensive stroke protocols.^[15]^ Diffusion-weighted imaging (DWI) is a technique used on the basis of MRI, which has higher sensitivity and accuracy for acute ischemia stroke which has been made clear through comparison with the other imaging methods.^[16]^ DWI is sensitive to acute cellular injury in cerebral ischemia and can be used in order to assess ischemic lesions in the first few hours.^[17]^ Despite the abundant amount of literature that analyze the various detection and therapeutic aspects of different imaging methods, no comprehensive literature that investigates the diagnostic values of ischemic stroke by ultrasonography, CT, and MRI through a network meta-analysis has been made available. Thus, this study aimed to compare diagnostic values among ultrasonography, CT and MRI by a network meta-analysis in the hopes of understanding which imaging method is best served to treat/diagnose acute ischemic stroke.

## 2 Materials and methods

### 2.1 Ethical statement

All 13 enrolled studies in this network meta-analysis have consented to the ethical statements provided to them in regards to the current investigation.

### 2.2 Literature search

An electronic search of English literature databases such as Cochrane Library, PubMed, Embase and China national knowledge internet (CNKI) and Wan-fang databases for articles that were published from the beginning of this investigation up until February 2019 was carried out. Literature was manually searched using different combinations of keywords and free words. The search terms included: stroke, MRI, CT, ultrasonography, DWI, and so on.

### 2.3 Inclusion and exclusion criteria

The inclusion criteria used to find literature were:

(1) Study design should be diagnostic tests studies.
(2) The imaging methods should include 2 or more of the following imaging methods: traditional CT, CTA, CTP, DWI, MRA, MRI, and ultrasonography.
(3) Subjects should be consecutive ischemic stroke patients aged between 18 and 100 years old.
(4) Outcomes of the research should include parameters, such as sensitivity, specificity, positive predictive value (PPV), negative predictive value (NPV), and accuracy.
(5) Gold standard, namely the final imaging and clinical examination, can result in a proper diagnosis.

The exclusion criteria were: studies with insufficient data integrity or those that were not related to ischemic stroke, repeated published literature, conference reports, systematic reviews or summary articles, non-English literature, and non-human research.

### 2.4 Data extraction and quality assessment

A standardized data extraction form was used in order to extract the relevant information required for this meta-analysis: the first author, published year, nationality, ethnicity, age, gender, the gold standard, sensitivity, specificity, NPV, PPV, and accuracy. This was carried out by 2 investigators who independently extracted and incorporated the data into this study. Disagreements regarding the extraction of the data were resolved when the discussion among several researchers reached a consensus. The quality of the included studies made by the two researchers was assessed according to the quality assessment of diagnostic accuracy studies (QUADAS-2).^[18]^ The QUADAS-2 includes the following domains: patient selection, reference standard, index text and flow of patients through the study/timing of index tests, and relevant reference standard. A Review Manager 5 (RevMan 5.2.3, Cochrane Collaboration, Oxford, UK) was used in order to assess both the quality and investigate publication bias.

### 2.5 Statistical analysis

Initially, we performed a direct comparison of the various kinds of treatment methods using a traditional pairwise meta-analysis. The results showed the pooled estimation between the odds ratio (ORs) and 95% credible intervals (CrIs) in strokes. Heterogeneity was analyzed by using a Chi-square test, with the value of *P*_*h*_ <.05 suggesting heterogeneity. In addition, an *I*^2^ statistic ^[19,20]^ was adopted in order to evaluate the degree of heterogeneity, with the range of *I*_2_ value between 0% and 100%. The higher the *I*_2_ value was, the more obvious the heterogeneity was among the specimen. The values of *P*_*h*_ <.05 or *I*_2_ >50% indicated that there was greater heterogeneity in the specimen. Based on the results, the random-effect model was used for further analysis, otherwise, a fixed-effect model was performed. Next, R 3.2.1 was used for drawing network diagrams, with each diagram node representing each intervention. The node size represented the sample size, as well as the line thickness between nodes represented the study numbers. Adding onto this, we made use of different comparisons involving interventions using a Bayesian network meta-analysis: the basis of each analysis was non-informative before gain-effect sizes and precision. After four chains and a 20,000-simulation burn-in stage, the convergence and subsequent lack of auto-correlation were examined and confirmed; finally, direct probability statements were obtained from an additional 50,000-simulation stage.^[21]^ We implemented the node-splitting method in order to estimate the consistency between the direct and indirect evidence for the whole study, then chose the consistency model or inconsistency model based on the results.^[22]^ To obtain a better interpretation of ORs, the probability of every intervention was calculated in order to find the most effective methods based on a Bayesian approach that employs probability values summarized as the surface under the cumulative ranking curve (SUCRA), the significance or difference in the larger the SUCRA value, the better the rank of the intervention.^[23,24]^ A cluster analysis was also adopted in order to evaluate the discrepancies the different imaging methods presented in the diagnosis of ischemic stroke. In other words, we can cluster different intervention measures according to the similarity between two specific variables. We can then use the advantages and disadvantages of different imaging methods in judging their overall effect.^[25]^ R (V.3.2.1) package gemtc (V.0.6) and Markov Chain Monte Carlo engine Open BUGS (V.3.4.0) are both used for making all necessary computations.

## 3 Results

### 3.1 Baseline characteristics of included studies

**Figure 1:**
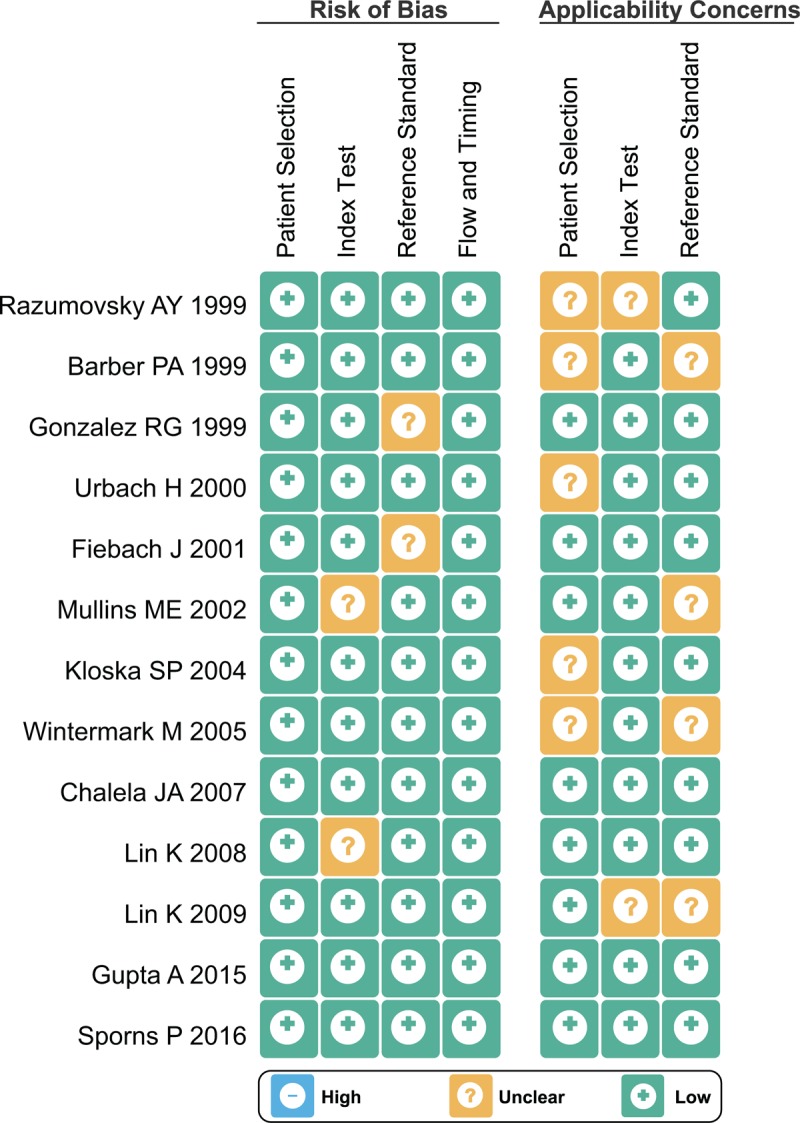
The QUADAS-2 rating scale for evaluating the quality of the included studies. QUADAS-2 = quality assessment of diagnostic accuracy studies.

We initially sought out and found 2676 voluntary candidate studies. After reviewing the titles and abstracts, we excluded the following: 42 studies as duplicates, 136 as letters or summaries, 286 as non-human studies, and 186 as non-English studies, leaving us with 2026 qualifying remaining articles. After a 2026 assessment of the remaining articles, we excluded an additional 496 case-control studies, 690 studies unrelated to ischemic stroke, 825 studies unrelated to the imaging methods, and 2 studies without either data resources or incomplete documentation. Ultimately, 13 diagnostic tests among the 1226 remaining articles were found eligible for this current network meta-analysis and were subsequently used for further analysis (Appendix Fig. 1).^[26–38]^ A total of 1462 patients with ischemic stroke were diagnosed by the gold criteria. The included studies related to the present study were published between 1999 and 2015. All of the subjects involved in the 13 studies were Caucasians. Six of the 13 studies were three-arm trials, with the remaining seven of them being two-arm trials. Baseline characteristics of the included studies are summarized as shown in Table 1. The Cochrane risk of bias assessment for all included studies is shown in Figure 1.

**Table 1:**
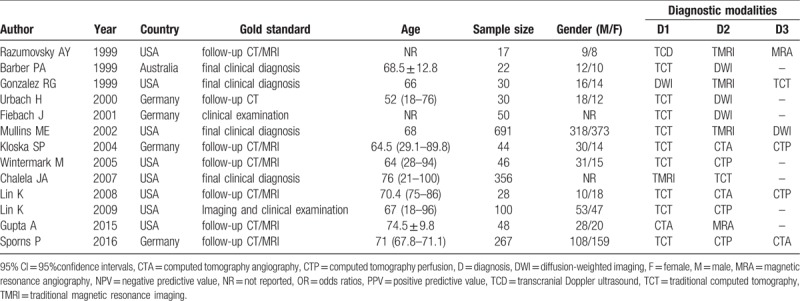
The baseline characteristics for included studies.

### 3.2 Pairwise meta-analysis of the eight imaging methods for the diagnosis of ischemic stroke

We performed a direct paired comparison of the seven imaging methods in the diagnosis of ischemic stroke. The CT methods include traditional CT, CTA, and CTP. The MRI methods include traditional MRI, DWI, and magnetic resonance angiography. Among CT methods, the traditional CT (sensitivity: OR = 0.16, 95% CI = 0.12∼0.21; NPV: OR = 0.30, 95% CI = 0.22∼0.41; accuracy: OR = 0.22, 95% CI = 0.17∼0.27) and CTA (sensitivity: OR = 4.49, 95% CI = 3.14∼6.41; NPV: OR = 2.87, 95% CI = 1.95∼4.22; accuracy: OR = 3.33, 95% CI = 2.41∼4.61) had a relatively low sensitivity, NPV, and accuracy than CTP. Among the MRI methods, traditional MRI (sensitivity: OR = 6.42, 95% CI = 1.72∼23.90; accuracy: OR = 6.50, 95% CI = 1.82∼23.21) showed higher sensitivity and accuracy than magnetic resonance angiography; and traditional MRI (sensitivity: OR = 0.11, 95% CI = 0.06∼0.21; NPV: OR = 0.21, 95% CI = 0.10∼0.47; accuracy: OR = 0.14, 95% CI = 0.08∼0.26) showed lower sensitivity, NPV and accuracy than DWI. As for ultrasonography, compared with magnetic resonance angiography, transcranial Doppler ultrasound showed higher sensitivity, and accuracy (sensitivity: OR = 29.17, 95% CI = 3.42∼248.48; accuracy: OR = 6.50, 95% CI = 1.82∼23.21) (Table 2).

**Table 2:**
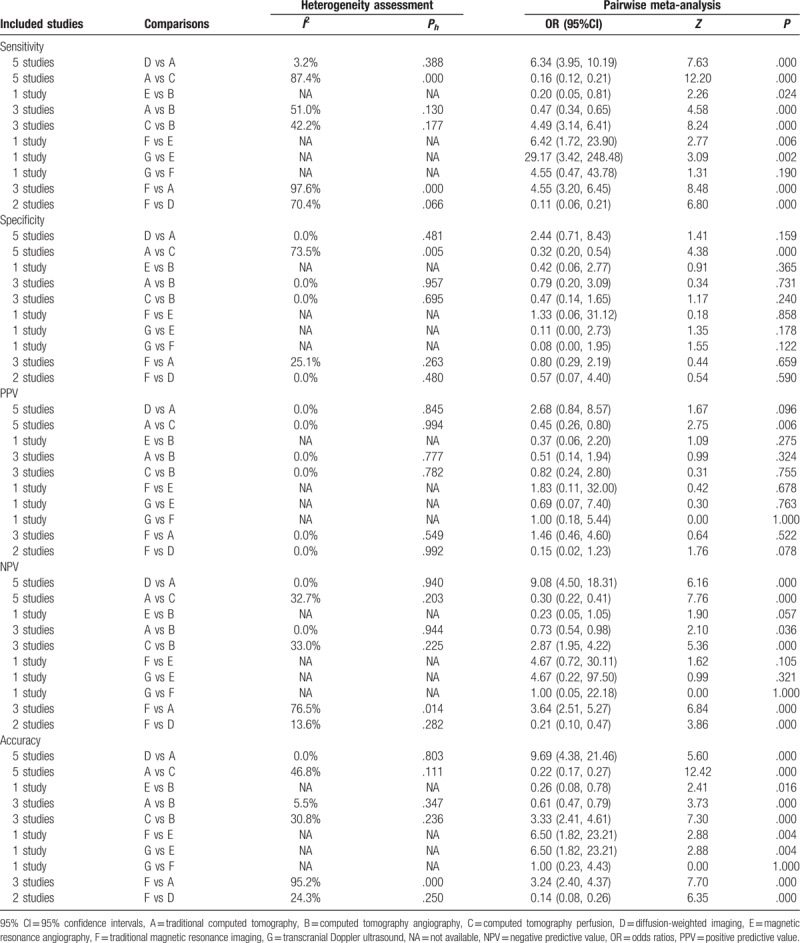
Estimated OR and 95%CI of pairwise meta-analysis for sensitivity, specificity, PPV, NPV, and accuracy for ischemia stroke.

### 3.3 Evidence network of eight imaging methods for the diagnosis of ischemic stroke

**Figure 2:**
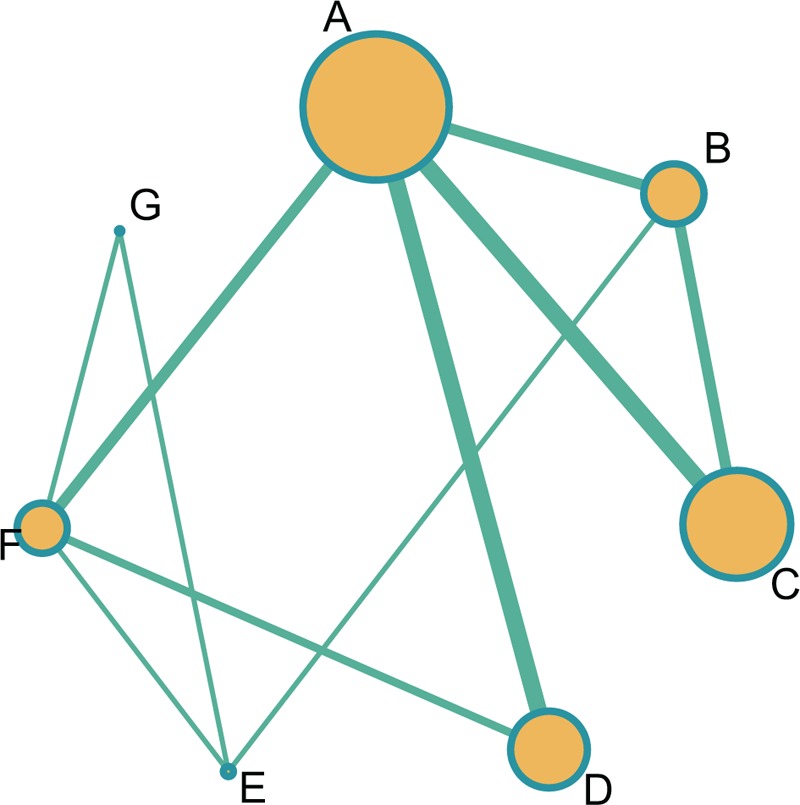
Evidence images of seven imaging methods for the diagnostic values of ischemic stroke. A = traditional computed tomography, B = computed tomography angiography, C = computed tomography perfusion, D = diffusion-weighted imaging, E = magnetic resonance angiography, F = traditional magnetic resonance imaging, G = transcranial Doppler ultrasound.

In terms of sensitivity, we can make the indication that both CT and CTP methods were used by a relatively large number of patients (Fig. 2).

### 3.4 Inconsistency test of seven imaging methods for the diagnosis of ischemic stroke

The results showed no inconsistencies among the studies thanks to a node-splitting method in terms of sensitivity, specificity, PPV, NPV, and accuracy (all *P* >.05) (Appendix Fig. 2–6). Therefore, due to the consistent nature of the results, logically the consistency model was applied.

### 3.5 Network meta-analysis of seven imaging methods for the diagnosis of ischemic stroke

**Figure 3:**
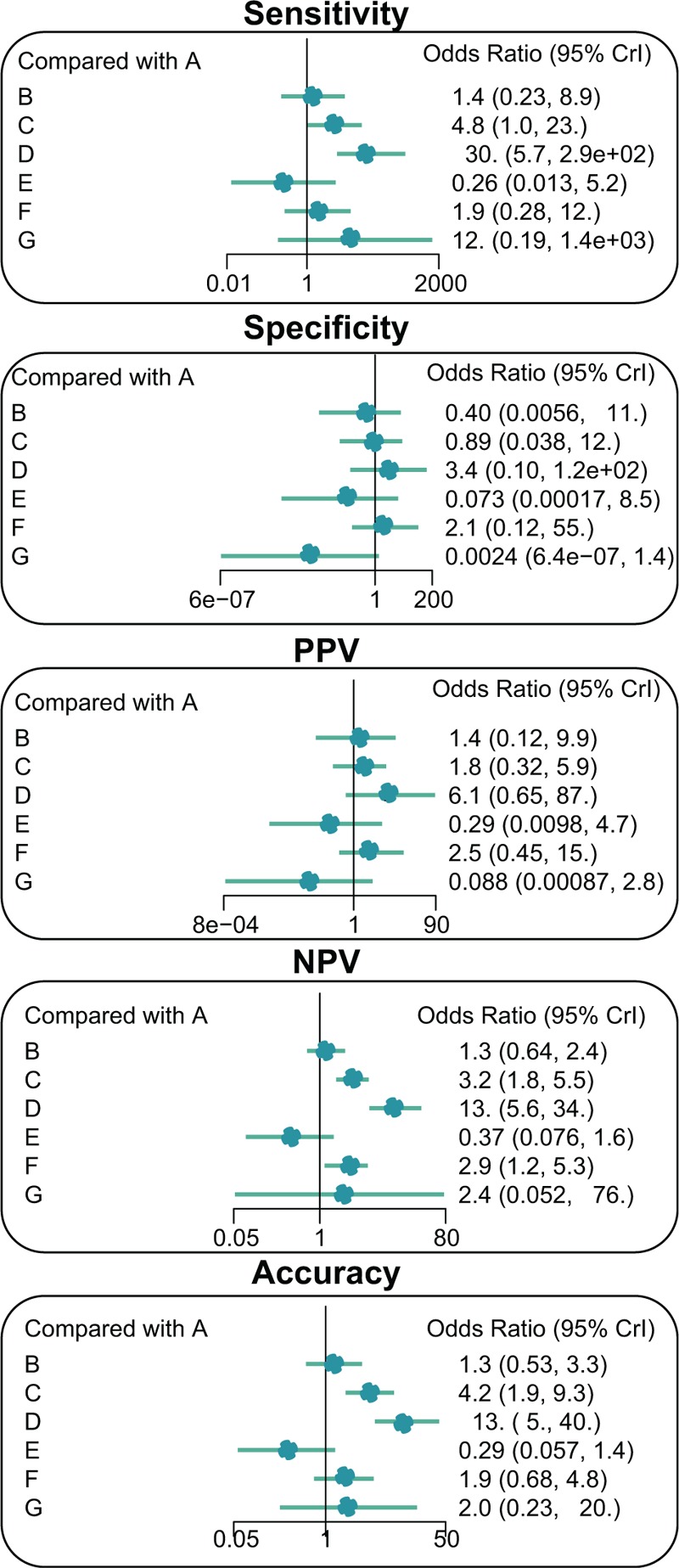
Forest plots representing the seven imaging methods for the diagnostic values of ischemic stroke. A = traditional computed tomography, B = computed tomography angiography, C = computed tomography perfusion, D = diffusion-weighted imaging, E = magnetic resonance angiography, F = traditional magnetic resonance imaging, G = transcranial Doppler ultrasound, NPV = negative predictive value, PPV = positive predictive value.

The results of this network meta-analysis demonstrated that DWI had a higher sensitivity when compared with those of the traditional CT, CTA, magnetic resonance angiography, and traditional MRI (OR = 30.0, 95% CI = 5.7∼2.9e+02; OR = 21.0, 95% CI = 2.0∼4.0e+02; OR = 1.1e+02, 95% CI = 4.6∼4.8e+03; OR = 15.0, 95% CI = 2.0∼2.3e+02, respectively). Additionally, DWI still managed to have a relatively higher NPV in comparison with the traditional CT, CTA, CTP, magnetic resonance angiography, and traditional MRI (OR = 13.0, 95% CI = 5.6∼34.0; OR = 10.0, 95% CI = 3.7∼33.0; OR = 4.1, 95% CI = 1.5∼13.0; OR = 36.0, 95% CI = 7.0∼2.2e+02; OR = 4.6, 95% CI = 1.9∼16.0, respectively). Furthermore, DWI still had a relatively higher accuracy when compared with the traditional CT, CTA, magnetic resonance angiography, and traditional MRI (OR = 13.0, 95% CI = 5.0∼40.0; OR = 9.4, 95% C I = 2.7∼42.0; OR = 43.0, 95% CI = 7.8∼3.1e+02; OR = 6.7, 95% CI = 2.3∼26.0, respectively) (Fig. 3 & Appendix Table 1–2). These results suggested that DWI had a relatively higher sensitivity, NPV, and accuracy in comparison to the other methods involved traditional CT, CTA, magnetic resonance angiography, and traditional MRI.

### 3.6 SUCRA values of seven imaging methods for the diagnosis of ischemic stroke

The SUCRA values of the seven imaging methods were summarized and shown in Table 3. As for the CT methods, CTP showed the higher SUCRA values (sensitivity: 71.93%; specificity: 64.69%; PPV: 67.34%; NPV: 73.53%; accuracy: 81.16%). As for the MRI methods, DWI showed the higher SUCRA values (sensitivity: 94.13%; specificity: 83.07%; PPV: 90.69%; NPV: 97.74%; accuracy: 98.72%). The SUCRA curves suggested that DWI showed better efficacy while CTP showed lower efficacy in the diagnosis of ischemic stroke.

**Table 3:**
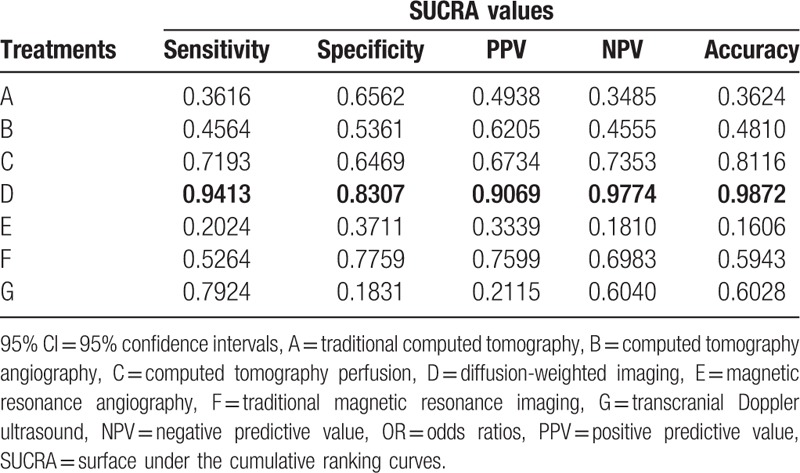
SUCRA values of seven diagnostic modalities under five endpoint outcomes.

### 3.7 Cluster analysis for the outcomes of sensitivity and accuracy for the diagnosis of ischemic stroke

**Figure 4:**
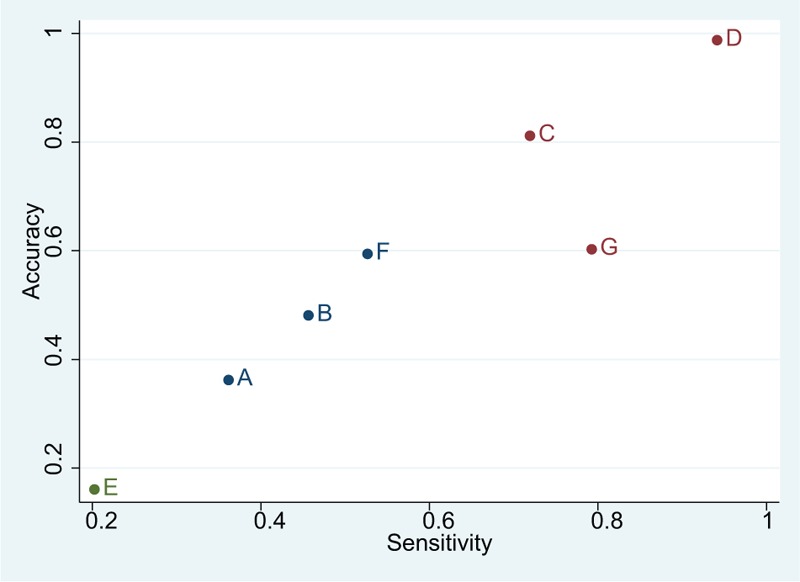
Cluster ranking plots of sensitivity and accuracy for the diagnostic values of ischemic stroke. A = traditional computed tomography, B = computed tomography angiography, C = computed tomography perfusion, D = diffusion-weighted imaging, E = magnetic resonance angiography, F = traditional magnetic resonance imaging, G = transcranial Doppler ultrasound.

The previously employed cluster analysis demonstrated that DWI had the highest diagnostic value for the detection of ischemic stroke in terms of both sensitivity and accuracy (Fig. 4).

### 3.8 Sensitivity analysis for the diagnosis of ischemic stroke

The sensitivity analysis indicated that the inclusion of literature in our study which may cause bias had little influence on both the specificity and PPV, but no effects on the final results (Table 4).

**Table 4:**
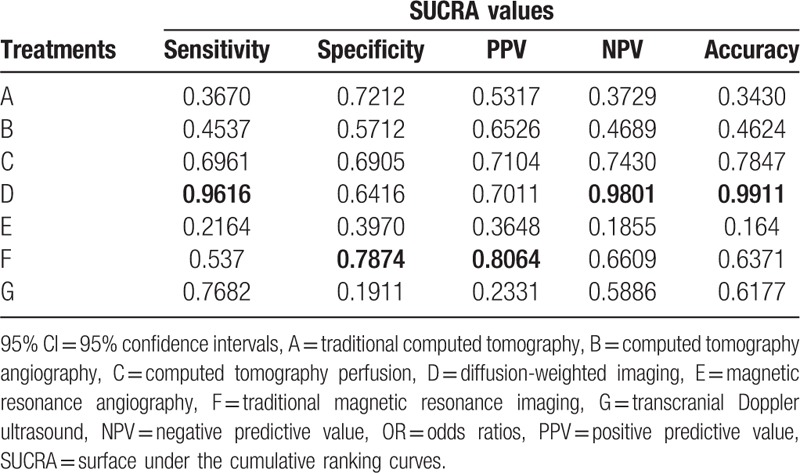
Sensitivity analysis of outcomes by excluding trials with a high risk of bias.

## 4 Discussion

We conducted a network meta-analysis in order to compare the diagnostic values of seven different imaging methods and their diagnostic values for ischemic stroke. We hope that our findings will assist physicians and doctors in choosing the most suitable imaging methods for the diagnosis of ischemic stroke.

The most important finding in our meta-analysis was that DWI had higher sensitivity, NPV, and accuracy. DWI is the optimal imaging technique for both the diagnosis and management of acute ischemic stroke.^[39]^ Despite the optimal imaging choice for detecting ischemic strokes, there is still only limited evidence available in regards to the value DWI has in the treatment/management of ischemic stroke with a low-field magnetic resonance system.^[40]^ The initial DWI lesion volume was demonstrated to be correlated with the final infarct volume as well as both neurological and functional outcomes, which could subsequently serve as an early prognostic tool.^[41]^ In addition, DWI can be used to accurately monitor the evolution of the ischemic core over time.^[40]^ Consistent with the results of our findings, Simonsen et al found that DWI had a sensitivity of 90.4% in the diagnosis of ischemic stroke, and an associated smaller study found that the false-negative rate was 5.8% when imaging was carried out within the first 48 hours.^[42]^ All patients who were eventually diagnosed with ischemic stroke also showed evidence of ischemia by DWI. On the contrary, 25% of patients with ischemic stroke had a normal acute CT scan within 6 hours of initial symptom onset.^[26]^ A recent study found that DWI determined the existence and location of the infarct, with a 73% sensitivity for detection of ischemic strokes that occurred within three hours of onset and a 92% sensitivity for the occurrence of strokes after a 12-hour onset. Oppositely, the sensitivity of CT for the same time period of detection for stroke after onset was found to be 12% and 16%, respectively.^[40]^ Another study found that the high b-value of DWI was better served for visualizing and detecting both small and multiple lesions.^[43]^ One of the advantages that DWI has shown over CT in the detection of acute ischemia is the significantly greater contrast-to-noise-ratio (CNR). Furthermore, a study showed that after 48 hours from the stroke onset, DWI showed a higher sensitivity in the detection of ischemic lesion of all clinical stroke subtypes than when compared to CT.^[44]^ When compared with a CT, DWI was found to be more accurate for the identification of acute infarction and showed a higher sensitivity for the detection of more than 33% MCA involvement.^[45]^ Consequently, the reliability and reproducibility of CT in detecting and estimating the degree of ischemic stroke change is controversial: it is not easy to distinguish early ischemic changes on CT.^[26]^ The accuracy of MRI perfusion-diffusion imaging remains superior to CTP, especially inaccurate identification of infarct core.^[46]^ MRI throughout our study has obviously shown higher accuracy, which can reduce security risks and provide a wider range of information than a standard CT.^[47]^ DWI has been shown to be the most effective and should be considered effective within 12 hours of the onset of symptoms for the diagnosis of acute ischemic stroke.^[48]^

The cluster analysis revealed that DWI had the highest diagnostic value for ischemic stroke in terms of sensitivity, NPV, and accuracy amongst the seven imaging techniques. The higher diagnostic value of DWI was on the basis of both different imaging methods and the increased edema over time.^[49]^ However, there were some disadvantages of DWI, which could not be ignored. On the one hand, DWI is incompletely sensitive to patients with only a perfusion lesion (“total mismatch”) and cannot be performed in certain patients that have metallic implants, agitation, or claustrophobia.^[50,51]^ Additionally, it had been reported that DWI usually takes a longer period of time than CT-based imaging to obtain an imaging. This can lead to potentially harmful results from treatment delay that may outweigh the benefits of a more precise diagnosis.^[52]^ Copen et al have demonstrated in a previous study that CTP-derived cerebral blood flow (CBF) maps cannot substitute for DWI in the measurement of the ischemic core, for DWI could detect ischemic lesions with a notably high rate.^[53]^ Another study also suggested that DWI detected more frequently than CT in terms of ischemic lesions in all aspects regions apart from the caudate head and internal capsule.^[54]^ A study has suggested that DWI is highly accurate for the diagnosis for stroke within 6-h symptom onset, which is superior to CT and conventional MRI.^[27]^ In addition, during the diagnosis of stroke in the early period, namely 12 hours after presentation, DWI is superior to conventional MRI and CT.^[31]^ Besides, it has been revealed that DWI should be utilized instead of CT as the approach in stroke imaging for the reason that DWI showed the infarct core in almost every patient with ischemic stroke and indicated the actual minimum range of infarction in almost every patient at the time of imaging.^[49]^ Furthermore, evidence has revealed that the detectability and detection rate of acute hemisphere infarcts are markedly higher with DWI than with CI.^[29]^ All these aforementioned evidence has confirmed that DWI is superior to other imaging methods in ischemic stroke.

In conclusion, the results of this network meta-analysis suggested that DWI showed the highest diagnostic value regarding ischemic stroke in comparison with all of the other imaging methods. Advantages of our study included the wide range of comparison, allowing us to compare all seven different imaging methods in order to assess the diagnostic values of ischemic stroke in patients. However, there existed some limitations in this study. Firstly, noncontrast CT was reported to be more cost-effective for its ability to detect intra-cerebral hemorrhage which will result in therapy adjustment. Although DWI showed higher sensitivity than noncontrast CT, further study is needed to determine the criteria for DWI cost-effective use so as to suggest which patients will benefit from DWI. Second, the inaccuracy of the rankings was evaluated but the true positions of the treatments were not assessed. Third, the present study did not evaluate other factors that might affect the reliability of rankings, like small-study effects across trials or the risk of bias within trials.^[55]^

## Acknowledgments

The authors express their sincerest appreciation to reviewers for their progressive feedback.

## Author contributions

**Conceptualization:** Xiao-Hong Zhang, Hui-Min Liang.

**Formal analysis:** Xiao-Hong Zhang, Hui-Min Liang.

**Funding acquisition:** Xiao-Hong Zhang, Hui-Min Liang.

**Investigation:** Xiao-Hong Zhang, Hui-Min Liang.

**Resources:** Hui-Min Liang.

**Supervision:** Xiao-Hong Zhang.

**Writing – original draft:** Xiao-Hong Zhang, Hui-Min Liang.

**Writing – review & editing:** Xiao-Hong Zhang, Hui-Min Liang.

## Supplementary Material

Supplemental Digital Content

## Supplementary Material

Supplemental Digital Content
