# Supplementary material for: Systematic review with network meta-analysis: Diagnostic values of ultrasonography, computed tomography, and magnetic resonance imaging in patients with ischemic stroke
Source: Medicine (Baltimore). 2019 Jul 26;98(30):e16360. doi: 10.1097/MD.0000000000016360 (PMC6709059; doi:10.1097/MD.0000000000016360)
Supplement: Supplemental Digital Content [file medi-98-e16360-s001.pdf]

PRISMA Flow Diagram

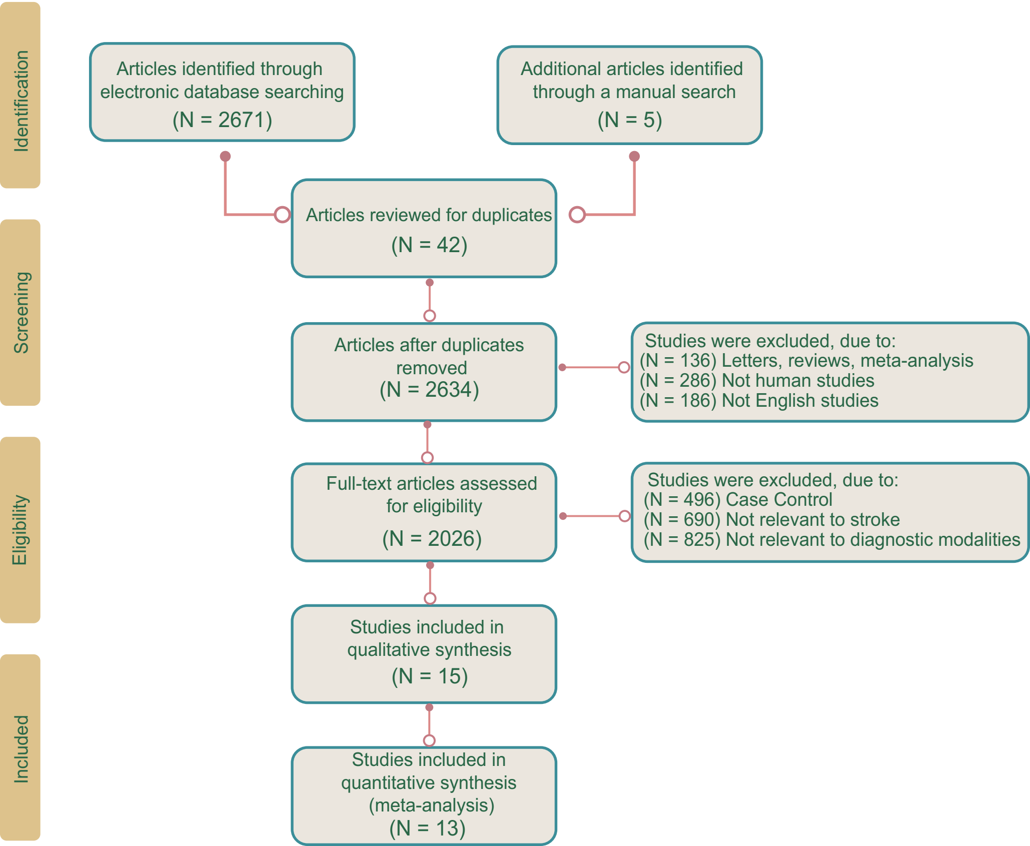

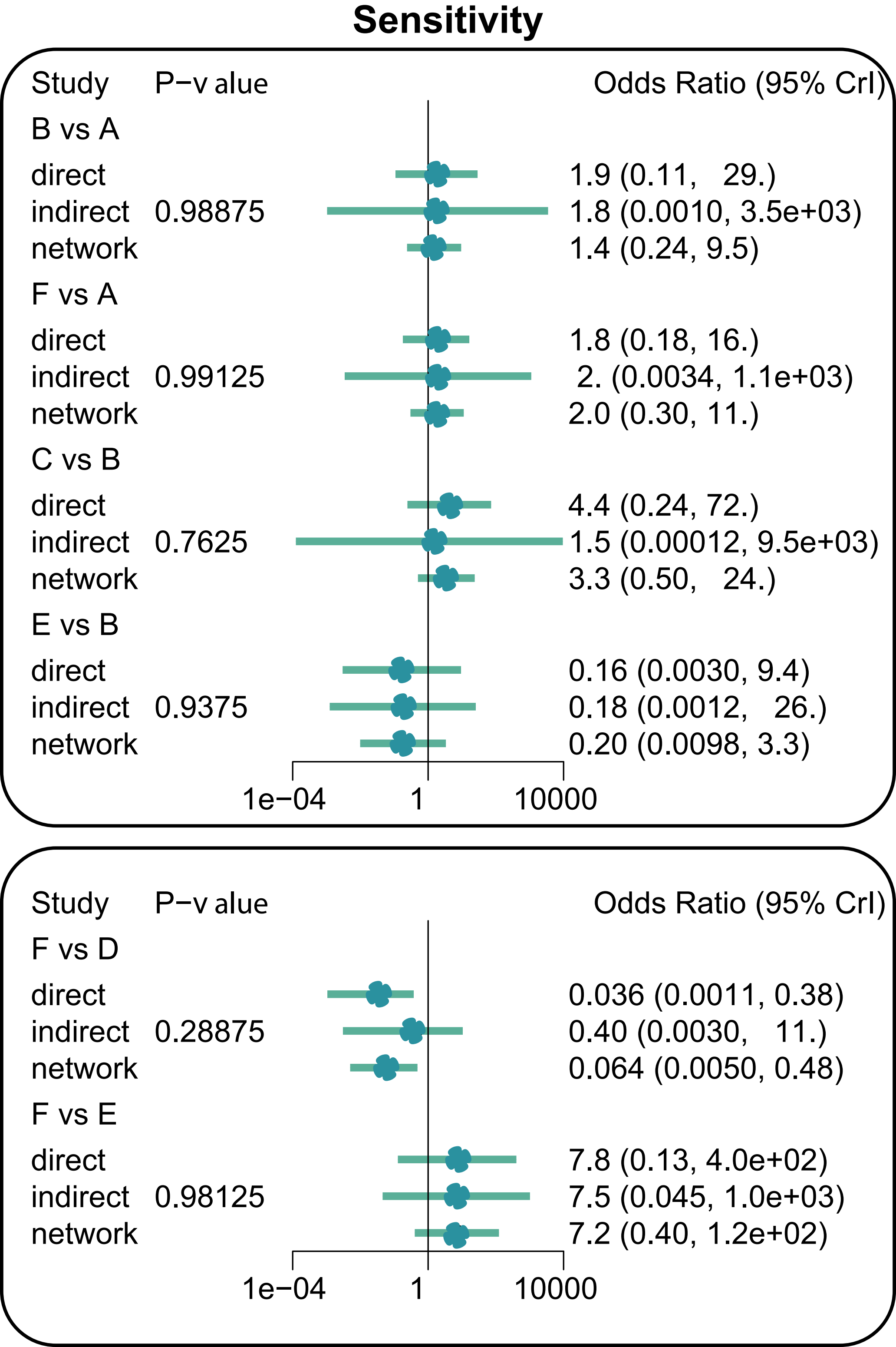

Sensitivity
Study
P−v alue
Odds Ratio (95% CrI)
B vs A
direct
1.9 (0.11, 29.)
indirect
0.98875
1.8 (0.0010, 3.5e+03)
network
1.4 (0.24, 9.5)
F vs A
direct
1.8 (0.18, 16.)
indirect
0.99125
2. (0.0034, 1.1e+03)
network
2.0 (0.30, 11.)
C vs B
direct
4.4 (0.24, 72.)
indirect
0.7625
1.5 (0.00012, 9.5e+03)
network
3.3 (0.50, 24.)
E vs B
direct
0.16 (0.0030, 9.4)
indirect
0.9375
0.18 (0.0012, 26.)
network
0.20 (0.0098, 3.3)
1e−04
1
10000
Study
P−v alue
Odds Ratio (95% CrI)
F vs D
direct
0.036 (0.0011, 0.38)
indirect
0.28875
0.40 (0.0030, 11.)
network
0.064 (0.0050, 0.48)
F vs E
direct
7.8 (0.13, 4.0e+02)
indirect
0.98125
7.5 (0.045, 1.0e+03)
network
7.2 (0.40, 1.2e+02)
1e−04
1
10000

Specificity

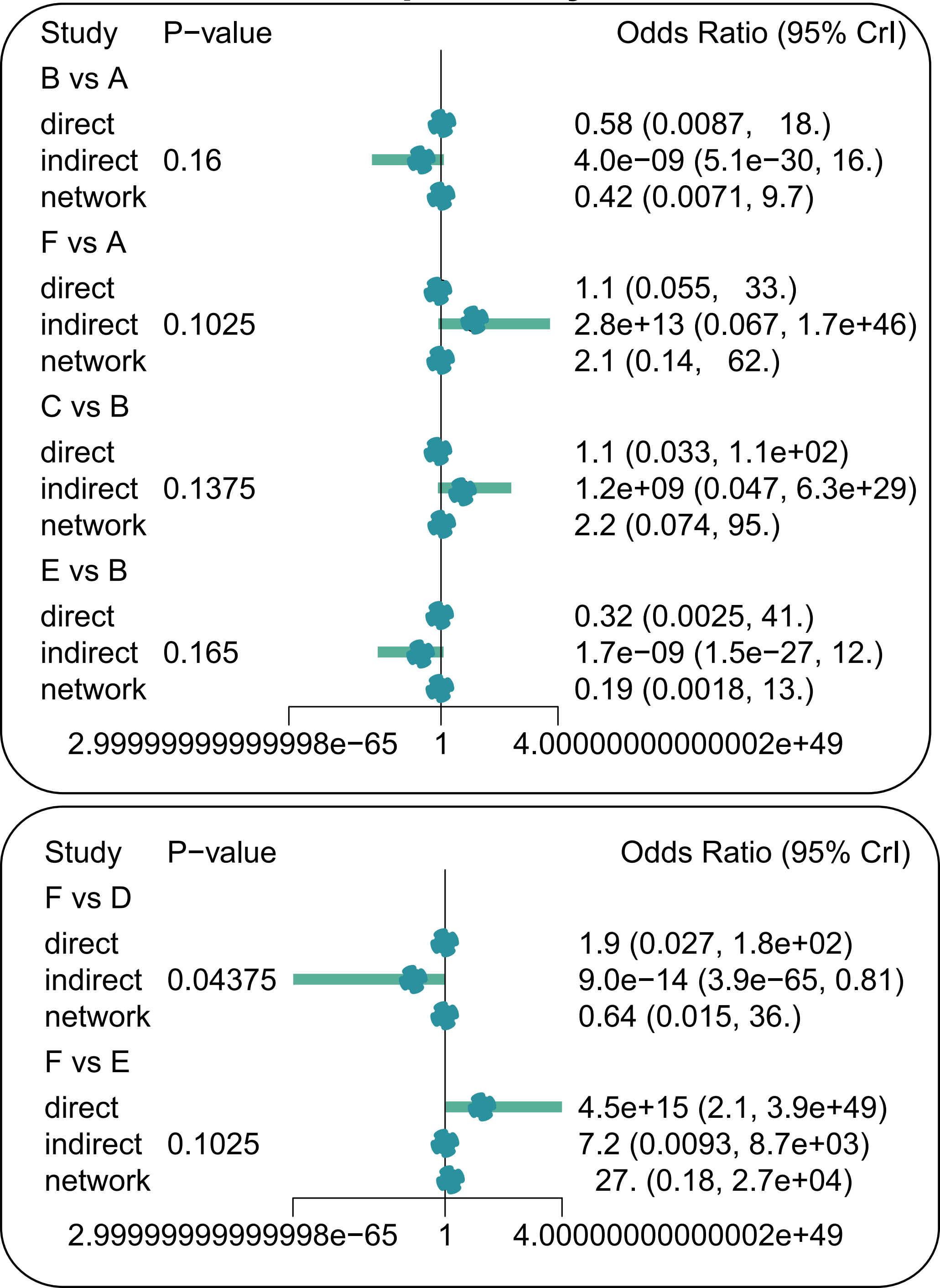

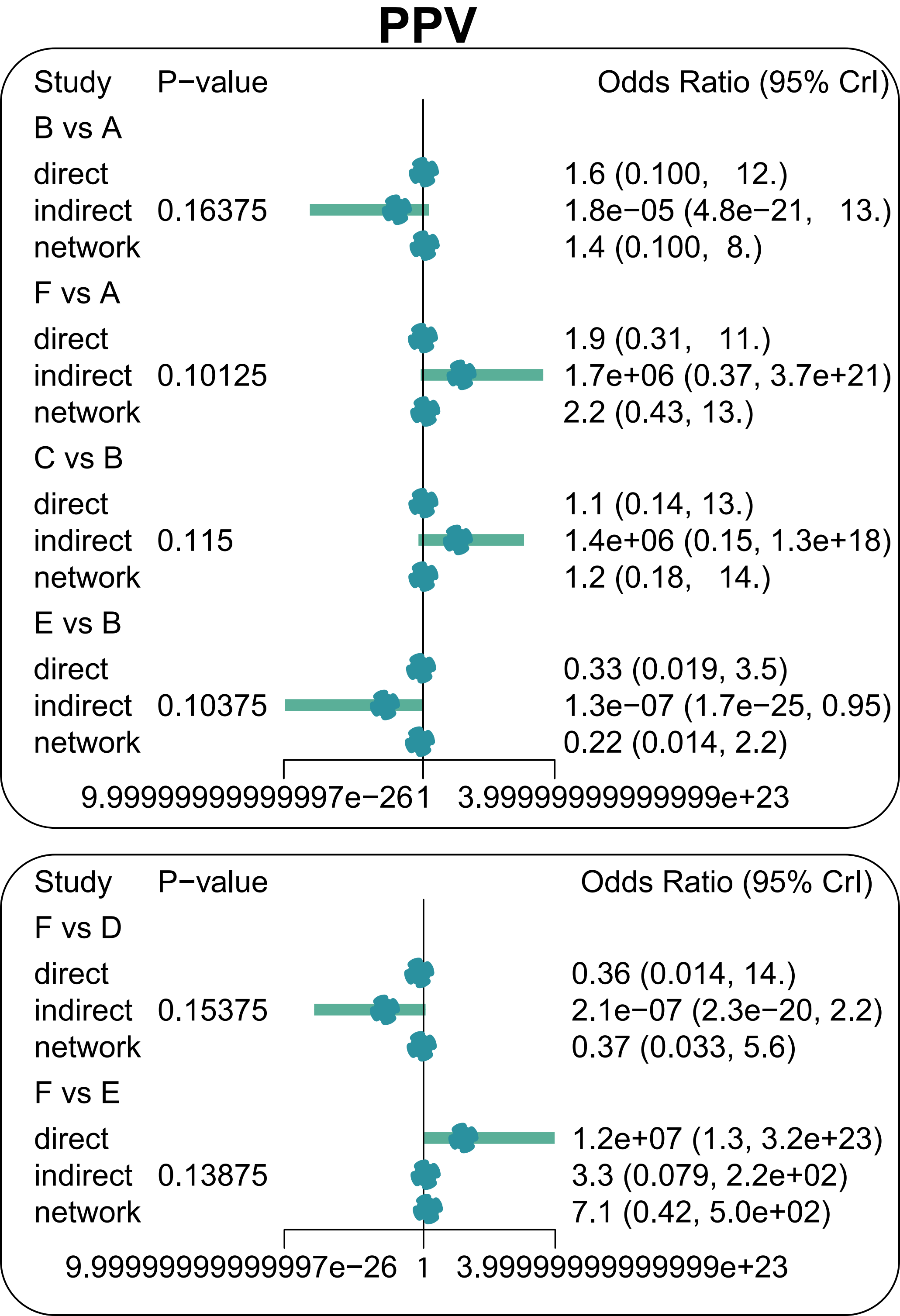
PPV
Study
P−value
Odds Ratio (95% CrI)
B vs A
direct
1.6 (0.100, 12.)
indirect
0.16375
1.8e−05 (4.8e−21, 13.)
network
1.4 (0.100, 8.)
F vs A
direct
1.9 (0.31, 11.)
indirect
0.10125
1.7e+06 (0.37, 3.7e+21)
network
2.2 (0.43, 13.)
C vs B
direct
1.1 (0.14, 13.)
indirect
0.115
1.4e+06 (0.15, 1.3e+18)
network
1.2 (0.18, 14.)
E vs B
direct
0.33 (0.019, 3.5)
indirect
0.10375
1.3e−07 (1.7e−25, 0.95)
network
0.22 (0.014, 2.2)
9.99999999999997e−26
1
3.99999999999999e+23
Study
P−value
Odds Ratio (95% CrI)
F vs D
direct
0.36 (0.014, 14.)
indirect
0.15375
2.1e−07 (2.3e−20, 2.2)
network
0.37 (0.033, 5.6)
F vs E
direct
1.2e+07 (1.3, 3.2e+23)
indirect
0.13875
3.3 (0.079, 2.2e+02)
network
7.1 (0.42, 5.0e+02)
9.99999999999997e−26
1
3.99999999999999e+23

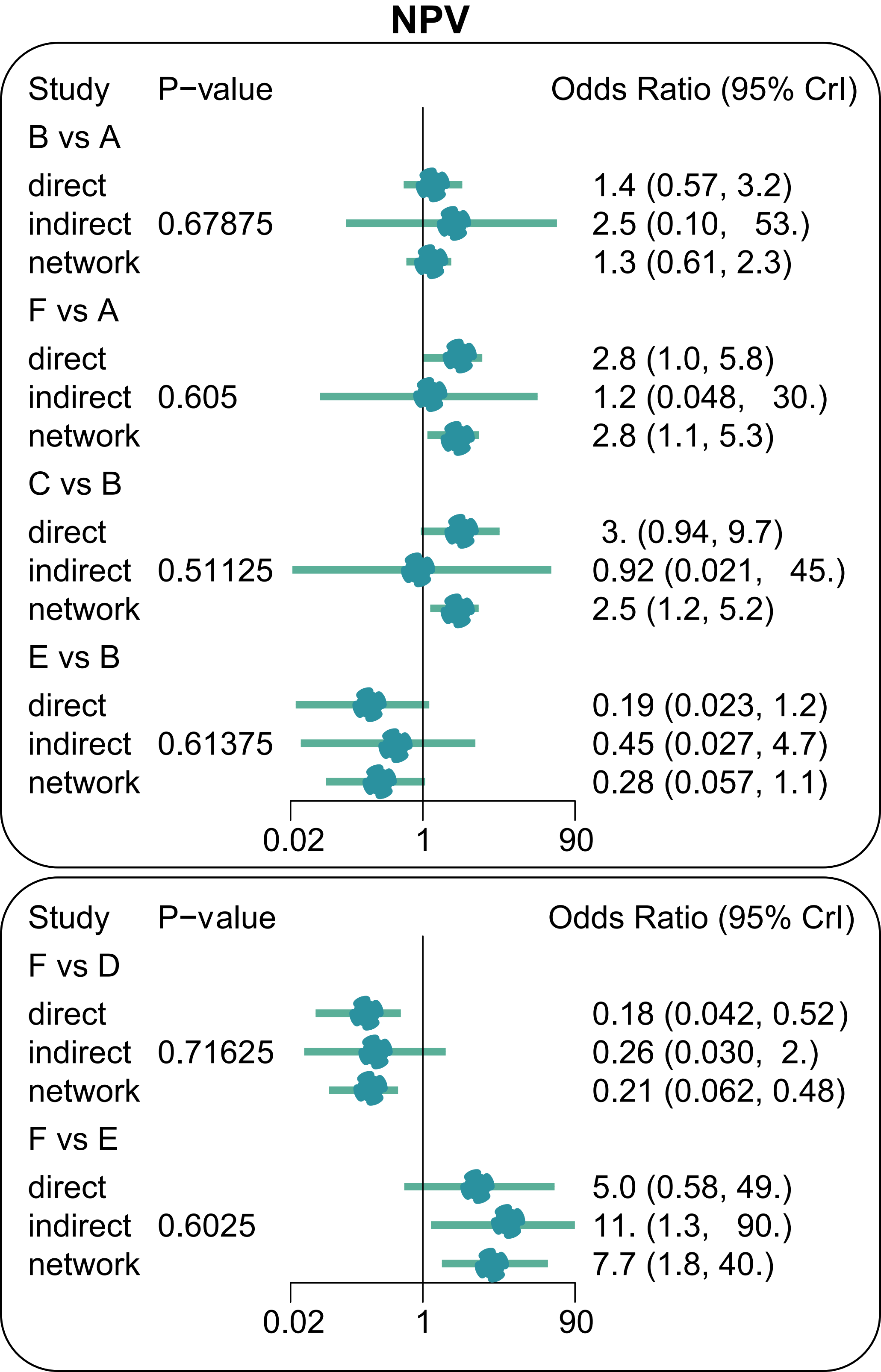
NPV
Study
P−value
Odds Ratio (95% CrI)
B vs A
direct
1.4 (0.57, 3.2)
indirect
0.67875
2.5 (0.10, 53.)
network
1.3 (0.61, 2.3)
F vs A
direct
2.8 (1.0, 5.8)
indirect
0.605
1.2 (0.048, 30.)
network
2.8 (1.1, 5.3)
C vs B
direct
3. (0.94, 9.7)
indirect
0.51125
0.92 (0.021, 45.)
network
2.5 (1.2, 5.2)
E vs B
direct
0.19 (0.023, 1.2)
indirect
0.61375
0.45 (0.027, 4.7)
network
0.28 (0.057, 1.1)
0.02
1
90
Study
P−value
Odds Ratio (95% CrI)
F vs D
direct
0.18 (0.042, 0.52)
indirect
0.71625
0.26 (0.030, 2.)
network
0.21 (0.062, 0.48)
F vs E
direct
5.0 (0.58, 49.)
indirect
0.6025
11. (1.3, 90.)
network
7.7 (1.8, 40.)
0.02
1
90

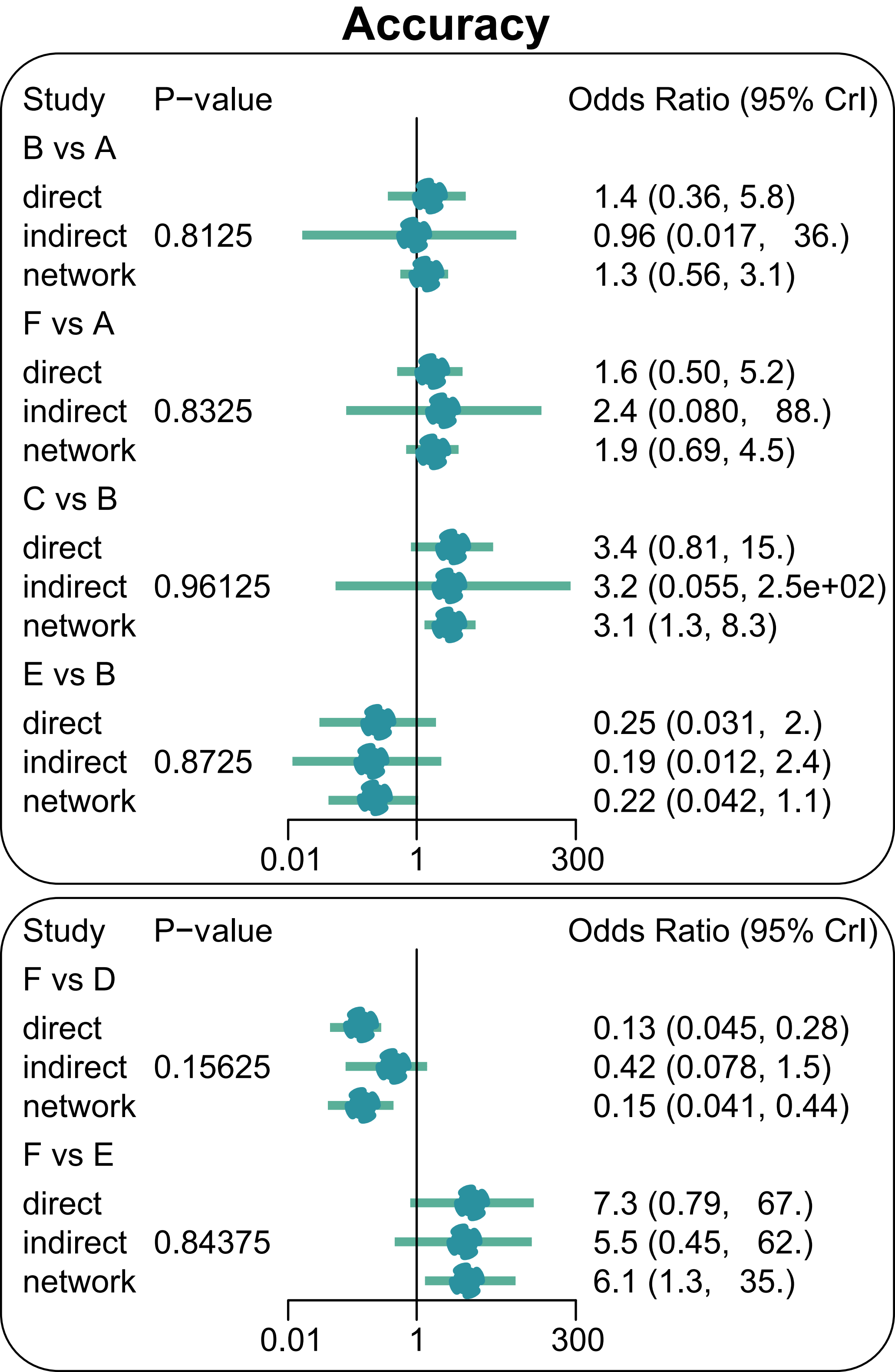
Accuracy
Study
P−value
Odds Ratio (95% CrI)
B vs A
direct
1.4 (0.36, 5.8)
indirect
0.8125
0.96 (0.017, 36.)
network
1.3 (0.56, 3.1)
F vs A
direct
1.6 (0.50, 5.2)
indirect
0.8325
2.4 (0.080, 88.)
network
1.9 (0.69, 4.5)
C vs B
direct
3.4 (0.81, 15.)
indirect
0.96125
3.2 (0.055, 2.5e+02)
network
3.1 (1.3, 8.3)
E vs B
direct
0.25 (0.031, 2.)
indirect
0.8725
0.19 (0.012, 2.4)
network
0.22 (0.042, 1.1)
0.01
1
300
Study
P−value
Odds Ratio (95% CrI)
F vs D
direct
0.13 (0.045, 0.28)
indirect
0.15625
0.42 (0.078, 1.5)
network
0.15 (0.041, 0.44)
F vs E
direct
7.3 (0.79, 67.)
indirect
0.84375
5.5 (0.45, 62.)
network
6.1 (1.3, 35.)
0.01
1
300

Appendix Figure 1. Flow chart showing literature search and study selection.

Appendix Figure 2. Node-splitting plot showing the sensitivity of the seven imaging methods for the diagnostic values of ischemic stroke. (A = traditional computed tomography; B = computed tomography angiography; C = computed tomography perfusion; D = diffusion-weighted imaging; E = magnetic resonance angiography; F = traditional magnetic resonance imaging; G = transcranial Doppler ultrasound)

Appendix Figure 3. The node-splitting plot showing the specificity of the seven imaging methods for the diagnostic values of ischemic stroke. (A = traditional computed tomography; B = computed tomography angiography; C = computed tomography perfusion; D = diffusion-weighted imaging; E = magnetic resonance angiography; F = traditional magnetic resonance imaging; G = transcranial Doppler ultrasound)

Appendix Figure 4. Node-splitting plot of PPV of the seven imaging methods for the diagnostic values of ischemic stroke. (A = traditional computed tomography; B = computed tomography angiography; C = computed tomography perfusion; D = diffusion-weighted imaging; E = magnetic resonance angiography; F = traditional magnetic resonance imaging; G = transcranial Doppler ultrasound; PPV = positive predictive value)

Appendix Figure 5. Node-splitting plot of NPV of the seven imaging methods for the diagnostic values of ischemic stroke. (A = traditional computed tomography; B = computed tomography angiography; C = computed tomography perfusion; D = diffusion-weighted imaging; E = magnetic resonance angiography; F = traditional magnetic resonance imaging; G = transcranial Doppler ultrasound; NPV = negative predictive value)

Appendix Figure 6. The node-splitting plot highlighting the accuracy of the seven imaging methods for the diagnostic values of ischemic stroke. (A = traditional computed tomography; B = computed tomography angiography; C = computed tomography perfusion; D = diffusion-weighted imaging; E = magnetic resonance angiography; F = traditional magnetic resonance imaging; G = transcranial Doppler ultrasound)
